# Post-COVID-19 syndrome. SARS-CoV-2 RNA detection in plasma, stool, and urine in patients with persistent symptoms after COVID-19

**DOI:** 10.1186/s12879-022-07153-4

**Published:** 2022-03-03

**Authors:** Francisco Tejerina, Pilar Catalan, Cristina Rodriguez-Grande, Javier Adan, Carmen Rodriguez-Gonzalez, Patricia Muñoz, Teresa Aldamiz, Cristina Diez, Leire Perez, Chiara Fanciulli, Dario Garcia de Viedma, Gregorio Marañon Microbiology ID COVID 19 Study Group, Luis Alcalá, Roberto Alonso, Beatriz Álvarez, Ana Álvarez-Uría, Alexi Arias, Luis Antonio Arroyo, Juan Berenguer, Elena Bermúdez, Emilio Bouza, Almudena Burillo, Ana Candela, Raquel Carrillo, Emilia Cercenado, Alejandro Cobos, Pilar Escribano, Agustín Estévez, Silvia Fernandez, Alicia Galar, Mª Dolores García, Paloma Gijón, Adolfo González, Helmuth Guillén, Jesús Guinea, Laura Vanessa Haces, Martha Kestler, Juan Carlos López, Carmen Narcisa Losada, Marina Machado, Mercedes Marín, Pablo Martín, Paloma Martín, Pedro Montilla, Zaira Moure, Patricia Muñoz, María Olmedo, Belén Padilla, María Palomo, Francisco Parras, María Jesús Pérez-Granda, Laura Pérez, Paula Pescador, Elena Reigadas, Cristina Rincón, Belén Rodríguez, Sara Rodríguez, Adriana Rojas, María Jesús Ruiz-Serrano, Carlos Sánchez, Mar Sánchez, Julia Serrano, Maricela Valerio, Mª Cristina Veintimilla, Lara Vesperinas, Teresa Vicente, Sofía de la Villa

**Affiliations:** 1 Clinical Microbiology and Infectious Diseases Department, Hospital General Universitario Gregorio Marañon, 46 C/ Doctor Esquerdo, 28009 Madrid, Spain; 2 Pharmacy Department, Hospital General Universitario Gregorio Marañón, 46 C/Dr Esquerdo, 28009 Madrid, Spain; 3 Instituto de Investigación Sanitaria Gregorio Marañón, Madrid, Spain; 4 Departamento de Medicina, Facultad de Medicina, Universidad Complutense de Madrid, Madrid, Spain; 5 Instituto de Salud Carlos III, Madrid, Spain; 6 CIBERES, CIBER Enfermedades Respiratorias, Madrid, Spain

## Abstract

**Background:**

There is a paucity of knowledge on the long-term outcome in patients diagnosed with COVID-19. We describe a cohort of patients with a constellation of symptoms occurring four weeks after diagnosis causing different degrees of reduced functional capacity. Although different hypothesis have been proposed to explain this condition like persistent immune activation or immunological dysfunction, to date, no physiopathological mechanism has been identified. Consequently, there are no therapeutic options besides symptomatic treatment and rehabilitation.

**Methods:**

We evaluated patients with symptoms that persisted for at least 4 weeks after COVID-19. Epidemiological and clinical data were collected. Blood tests, including inflammatory markers, were conducted, and imaging studies made if deemed necessary. Severe acute respiratory syndrome coronavirus 2 (SARS-CoV-2) reverse transcription polymerase chain reaction (RT-PCR) in plasma, stool, and urine were performed. Patients were offered antiviral treatment (compassionate use).

**Results:**

We evaluated 29 patients who reported fatigue, muscle pain, dyspnea, inappropriate tachycardia, and low-grade fever. Median number of days from COVID-19 to positive RT-PCR in extra-respiratory samples was 55 (39–67). Previous COVID-19 was mild in 55% of the cases. Thirteen patients (45%) had positive plasma RT-PCR results and 51% were positive in at least one RT-PCR sample (plasma, urine, or stool). Functional status was severely reduced in 48% of the subjects. Eighteen patients (62%) received antiviral treatment. Improvement was seen in most patients (p = 0.000) and patients in the treatment group achieved better outcomes with significant differences (p = 0.01).

**Conclusions:**

In a cohort of COVID-19 patients with persistent symptoms, 45% of them have detectable plasma SARS-CoV-2 RNA. Our results indicate possible systemic viral persistence in these patients, who may benefit of antiviral treatment strategies.

## Introduction

On March 11, 2020, the World Health Organization declared coronavirus disease 2019 (COVID-19) a global pandemic. By August 20, more than 20 million cases had been diagnosed and at least 750,000 deaths reported [1].

During the 2003 epidemic of severe acute respiratory syndrome (SARS), a large number of survivors reported persistent symptoms that included muscle weakness and pain, fatigue, and dyspnea. In some patients, these symptoms remained for several years and resembled those of chronic fatigue syndrome [2–4]. Moreover, functional status in these subjects was reduced, and in some cases, the patient was unable to return to work [5]. The etiological cause of these symptoms remains unknown, although hypothalamic/hypophysis dysfunction inducing hypocortisolism was proposed [6].

SARS and SARS-CoV-2 bind to the angiotensin-converting enzyme 2 receptor (ACE2) to enter the cells [7, 8]. Moreover, SARS-CoV-2 appears to have a stronger binding affinity to ACE2 than SARS [9]. The ACE2 receptor is widely distributed in multiple human tissues, expressed in lungs, cardiovascular system, kidneys, and gut [10]. This capacity to bind to a cellular receptor widely expressed in different organs may explain the systemic symptoms and clinical events described for COVID-19. SARS-CoV-2 viremia has been verified in critically ill patients; and it can be considered as an independent mortality predictor in these group of patients [11].

Many patients who have recovered from COVID-19 are reporting persistent symptoms similar to the ones described after the SARS epidemic. These symptoms not only occur in patients who have survived severe COVID-19 but are also being reported in patients who have suffered mild COVID-19 [12]. Tools for measuring the functional status of COVID-19 patients are being tested [13]. Persistent symptoms following discharge have been described in series of hospitalized COVID-19 patients, although studies to evaluate an etiological cause have not been carried out [14, 15]. Viremia in different respiratory virus like influenza 5 (H5N1) and rhinovirus has already been described [16, 17] and SARS-CoV-2 RNA associated to plasma exosomal vesicles has been described in non-critical COVID-19 patients [18]. To the best of our knowledge, positive SARS-CoV-2 RNA detection in extra-respiratory samples weeks after COVID-19 has not been described. SARS-CoV-2 infection can affect different organs and could cause persistent systemic viremia. Plasma, stool and urine could be a good subrogate sample to evaluate the possibility of systemic viremia.

Here, we describe a cohort of COVID-19 patients with persistent symptoms at least 4 weeks after diagnosis that present SARS-CoV-2 RNA in extra-respiratory samples.

## Methods

### Study population

COVID-19 positive patients confirmed by microbiological testing (nasopharyngeal RT-PCR and/or serological assays) or a combination of clinical symptoms and imaging tests, with persistent symptoms for at least 4 weeks after diagnosis were evaluated between May 6 and June 30, 2020, at the infectious disease unit of *Hospital General Universitario Gregorio Marañón* (a tertiary University Hospital in Madrid, Spain). Patients showed functional limitations, severe in some cases, and some were unable to return to work. The research ethics committee of the Hospital General Universitario Gregorio Marañón approved the study protocol.

### Clinical evaluation and data collection

Epidemiological characteristics were recorded including comorbidities, date of COVID-19 diagnosis, and clinical course. Acute COVID-19 was categorized as mild (patient did not develop pneumonia and was not hospitalized), moderate (developed pneumonia and was not hospitalized), or severe (developed pneumonia and was hospitalized). Antiviral SARS-CoV-2 treatment records, including glucocorticoids, were obtained.

The symptoms of the patients were recorded and their functional disability using a non-standard scale was classified as follows: Grade 0, patient had no symptoms; Grade I, patient had returned to work, performed daily duties, and had low-intensity persistent symptoms; Grade II, patient was able to work, performed daily activities, and had high-intensity symptoms; Grade III, patient was unable to work, performed basic duties; Grade IV, patient had not been able to return to work and could not perform daily tasks or needed help. Patients categorized as Grades III and IV were considered as having severe functional disability, Grade II moderate, and Grade I mild.

Blood tests included the following: hemogram, liver and kidney function tests, thyroid function test, inflammatory markers, and SARS-CoV-2 IgG nucleocapsid serology assay (ABBOT, CLIA Architect). Imaging tests including echocardiogram, chest X-ray, and thorax computed tomography with contrast were performed if needed for the evaluation.

On the first visit, SARS-CoV-2 reverse transcription polymerase chain reaction (RT-PCR) was performed in plasma, stool, and urine, and repeated every 7 days if positive, or if deemed necessary by the physician. Patients who had been diagnosed by RT-PCR from nasopharyngeal specimens, for whom a negative result had not been obtained, were retested (nasopharyngeal samples) during evaluation until negative on two consecutive occasions.

For nasopharyngeal specimens we used the diagnostic method implemented in our laboratory as follows: RNA extraction was carried out using the KingFisher (Thermo Fisher Scientific, USA) or the EasyMag (Biomeriuex, France) systems. Next, an RT-PCR was performed with the TaqPath COVID-19 CE-IVD rt-PCR kit (Thermo Fisher Scientific, USA). RNA extractions from urine and stool samples were done with EasyMag, and extractions from blood with EasyMag or the EZ1 system (Qiagen, Germany). RT-PCR in extra-respiratory samples detecting genes N and O were performed with the Novel Coronavirus (2019-nCoV) Nucleic Acid Diagnostic Kit (Sansure Biotech, China), as it proved to have the highest sensitivity among those evaluated in our laboratory throughout the pandemic. 20 µL of purified RNA were used as template for the RT-PCR. Samples were considered positive if the cycle threshold (Ct) value was ≤ 40, as specified by the manufacturer.

### Treatment

Patients were offered treatment with lopinavir/ritonavir and hydroxychloroquine (local guidelines of compassionate use) when they reported symptoms were causing a relevant impairment even if mild in the functional scale.

### Outcomes

Follow-up was done for at least 6 weeks after the first evaluation, regardless of whether the subject had received treatment. Improvement of the functional status was considered positive if the patient reported at least a one-point decrease in the functional scale in comparison to basal functional status. Degrees of clinical improvement were measured depending on the number of points of decrease in functional scale.

### Statistical analysis

Descriptive statistics were calculated: medians (interquartile ranges) and proportions. Association between variables was assessed with the Chi-squared test for linear trend and the ANOVA test. The SPSS statistical software v26.0 was used for the analyses.

## Results

### Baseline characteristics

Thirty patients were evaluated. One was excluded from the analysis after being diagnosed with Graves’ disease on the first visit.

Table 1 summarizes the epidemiological and clinical characteristics of the 29 patients included in the analysis. Sixty-two per cent of the subjects were female and median age was 45 years (36–56). Comorbidities were infrequent. Two patients were immunosuppressed (one HIV-positive and one diagnosed with Hodgkin’s lymphoma who had received rituximab). At diagnosis of COVID-19, half of the subjects (51.7%) had pneumonia and 24% had severe COVID-19. One patient was admitted to the intensive care unit but did not require mechanical ventilation. Nineteen patients (65%) received antiviral treatment, from which 78% were administered a combination of lopinavir/ritonavir and hydroxychloroquine. Median duration of lopinavir/ritonavir and hydroxychloroquine treatment in mild/moderate patients was 5 days. All patients with moderate/severe COVID-19 received antiviral treatment and two were treated with glucocorticoids.

**Table 1:** Baseline characteristics of study patients

	N = 29
Median age (IQR)—years	45 (36–56)
Female gender—n (%)	18 (62)
Comorbidities—n (%)	
Hypertension	1 (3.4%)
Dyslipidemia	3 (10.3%)
Obesity	3 (10.3%)
Immunosuppression	2 (6.8%)
Pneumonia COVID-19—n (%)	15 (51.7%)
COVID-19 severity—n (%)	
Mild	16 (55%)
Moderate	6 (20%)
Severe	7 (24%)
Antiviral treatment—n (%)	19 (65%)
COVID-19 severity and having received antiviral treatment—n (%)	
Mild	6 (37.5%)
Moderate	6 (100%)
Severe	7 (100%)
Type antiviral treatment—n (%)	
lpv/r	15 (78%)
HCQ	19 (100%)
Azm	3 (15%)
lpv/r + HCQ	15 (78%)
HCQ + Azm	3 (15%)
Glucocorticoids	2 (10%)

*IQR* interquartile range, *n* number, *lpv/r*  lopinavir/ritonavir, *HCQ* hydroxychloroquine, *Azm* Azithromycin

### Post-COVID-19 syndrome

Main symptoms and signs reported by patients were fatigue, muscle pain, dyspnea, inappropriate sinus tachycardia, and low-grade fever. Fatigue (86%) and muscle pain (62%) were the most frequently reported. More than a half of the subjects communicated having at least three symptoms. Severe functional disability and moderate to severe limitation was seen in 48% and 75% of the patients, respectively, with no relation between age or gender and the grade of functional limitation (p > 0.05) (Table 2).

**Table 2:** Clinical characteristics of post-COVID-19 syndrome

	N = 29
Symptoms—n (%)	
Fatigue	25 (86%)
Muscle pain	18 (62%)
Dyspnea	14 (48%)
Inappropriate sinus tachycardia	9 (31%)
Low-grade fever	9 (31%)
Functional status	
Grade 0	0 (0%)
Grade I—n (%)	7 (24%)
Grade II—n (%)	8 (27.5%)
Grade III—n (%)	5 (17.2%)
Grade IV—n (%)	9 (31%)
Median number of days until evaluation days (IQR)	57 (42–71)
Median number of days to extra-respiratory positive RT- PCR (IQR)	55 (39–67)
SARS-CoV-2 IgG tests—n (%)	25 (86%)
Hemoglobin (g/dL)—median (IQR)	14.3 (13.2–15)
Lymphocyte count (× 10E3/µl)—median (IQR)	1.4 (1.2–1.7)
Platelet count (× 10E3/µL)—median (IQR)	202 (184–238)
Serum creatinine (mg/dL)—median (IQR)	0.72 (0.63–0.82)
Alanin aminotransferase (U/L)—median (IQR)	24 (20–39.5)
> 40 U/L—n (%)	7 (25%)
≤ 40 U/L—n (%)	21 (75%)
Ferritin (µg/L)—median (IQR)	100 (38.5–204)
> 205 (µg/L)—n (%)	7 (25%)
≤ 205 (µg/L)—n (%)	21 (75%)
Positive SARS-CoV-2 RT-PCR	
Plasma—n (%)	13 (44.8%)
Stool—n (%)	5 (17.2%)
Urine—n (%)	4 (13.7%)
At least one positive result—n (%)	15 (51.7%)
Two positive results—n (%)	5 (17,2%)
Three positive results—n (%)	2 (6.8%)

*n * number, *IQR*  interquartile range

Median number of days from COVID-19 diagnosis to evaluation was 57, and 55 (39–67) from diagnosis to the first SARS-COV-2 RT-PCR test in specimens other than respiratory samples. IgG SARS-COV-2 antibodies were found in 85% of the patients. Four patients for whom a negative RT-PCR had never been obtained in nasopharyngeal swabs were positive on respiratory samples (at least 7 days before the first evaluation).

Five patients (17%) exhibited lymphopenia. Renal function was normal in all patients except in one with chronic renal failure. There were no remarkable increases in C-reactive protein and d-dimer levels. Slight increase of ferritin and ALT levels were observed in 25% of the patients. Imaging results in patients who underwent these tests did not reveal any pathological findings.

Initial RT-PCR was positive in half of the patients (51.7%) in at least one plasma, stool, or urine determination and in 13 patients (45%) in plasma. Five patients (17%) were positive in at least two determinations. Median Ct values in plasma, stool, and urine were 36 (35–37), 34 (31–39), and 37 (35–38), respectively. We found no association between the presence of positive SARS-CoV-2 RNA in extra-respiratory samples and worst functional disability (p = 0.425), in fact, four patients with mild functional disability had SARS-CoV-2 RNA in plasma on first evaluation.

Negative serology was found in four patients, all of them immunocompetent patients. Two had been diagnosed of COVID-19 by RT-PCR on nasopharyngeal swabs and the other two by clinical symptoms and imaging studies. SARS-CoV-2 infection was microbiologically confirmed in the two latter cases by RT-PCR in plasma or stool.

Among the patients for whom positive results were obtained by plasma RT-PCR, six (46%) had undergone mild clinical course of COVID-19 and three (23%) moderate.

### Antiviral treatment

Eighteen (62%) patients received antiviral treatment, 12 of which presented severely reduced functional status on first evaluation (Table 3). They were given lopinavir/ritonavir for at least 14 days, and 15 subjects received 200 mg of hydroxychloroquine concomitantly twice a day. Median duration of lopinavir/ritonavir treatment was 21 days. The duration of lopinavir/ritonavir treatment differed among patients as we gained more experience. Based on new scientific data, local guidelines on compassionate treatment withdrew the use of hydroxychloroquine and consequently the administration was interrupted. Eight patients received a 14-day lopinavir/ritonavir treatment and in the rest, it varied between 21 and 28 days.

**Table 3:** Antiviral treatment

	Received treatment (N = 18)	Untreated (N = 11)
Media age (IQR)—years	47.5 (45–55.7)	39 (35–53)
Female sex—n (%)	13 (72.2%)	5 (45.4%)
Covid-19 pneumonia—n (%)	11 (61.1%)	4 (36.3%)
Covid-19 severity—n (%)		
Mild	5 (27.7%)	2 (18.1%)
Moderate	4 (22.2%)	2 (18.1%)
Severe	9 (50%)	7 (63.6%)
COVID-19 acute antiviral treatment—n (%)	13 (72.2%)	6 (54.5%)
Positive results of plasma PCR—n (%)	9 (50%)	4 (36%)
At least one positive PCR—n (%)	10 (55.5%)	5 (45.4%)
Basal functional status		
Grade 0—n (%)	0 (0%)	0 (0%)
Grade I—n (%)	1 (5.5%)	6 (54.54%)
Grade II—n (%)	5 (27.7%)	3 (27.27%)
Grade III—n (%)	4 (22.2%)	1 (9%)
Grade IV—n (%)	8 (44.4%)	1 (9%)

*n*  number

RT-PCR in plasma was positive in half of the patients who initiated treatment (nine subjects); in these nine patients, no SARS-CoV-2 RNA was detected in plasma during the 1st week after starting the treatment. Four patients had positive RT-PCR in plasma on first evaluation and did not receive treatment; in one, SARS-CoV-2 RNA in plasma was found for 3 consecutive weeks, while negative results were obtained in the other three patients over the 1st week. There were also four patients with RT-PCR positive on nasopharyngeal swabs, three of which received treatment, after which RT-PCR results were negative.

No treatment-related major adverse events were observed. Three patients discontinued the treatment with hydroxychloroquine because of nausea and dizziness. Two patients interrupted treatment with lopinavir/ritonavir: one with previous paclitaxel-induced neuropathy after reporting increased numbness, and the other due to self-reported abnormal ocular movements. All side effects reversed on discontinuation of the drug.

### Outcomes

**Fig. 1:**
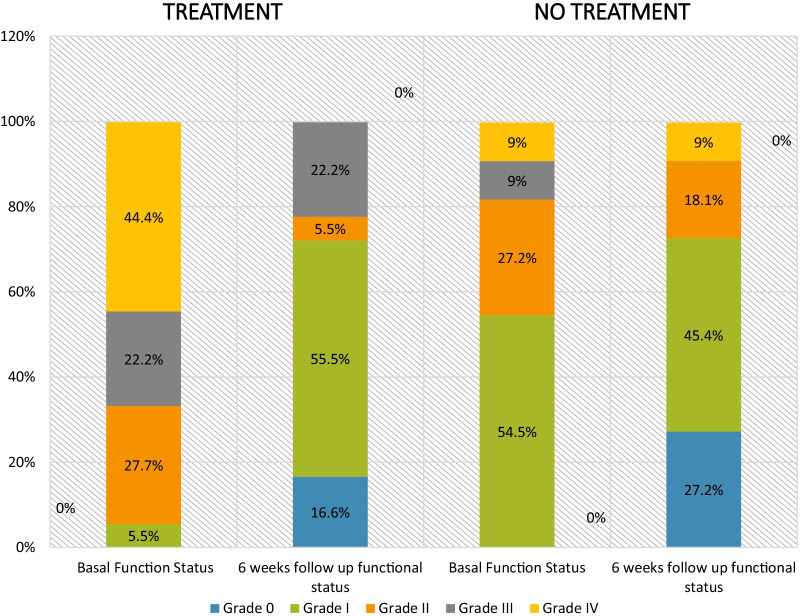
Outcomes of functional status

Overall, most patients (80%) showed positive changes in their functional status, with at least a one-point decrease in the functional scale. Improvement was seen in all patients who underwent treatment and in 55% with variation in two or more points in the disability scale. Six patients (54%) who did not receive treatment improved in one point, while no change in the functional limitation was seen in the other five untreated subjects (Fig. 1).

Recurrence of symptoms was seen in four positive plasma RT-PCR patients for whom functional improvement was observed while on treatment. Results of their RT-PCR in plasma remained negative.

## Discussion

There is a paucity of knowledge on the long-term outcome in patients diagnosed with COVID-19. To date, focus has been on understanding the acute course of the disease. However, as the number of infections grows, many patients are seeking medical care due to persistent symptoms, which, in some cases continues for several weeks leading to functional limitations that reduce patient’s quality of life. This medical condition where patients persist with a constellation of unspecific symptoms al least 4 weeks after COVID-19 has been recognized and denominated as Long COVID or Post-COVID-19 syndrome.

Here, we present the results from a small cohort of Long COVID patients. Many of the patients included in this study did not have a severe COVID-19 and most did not require hospitalization. Thus, the functional limitations in these patients differ from subjects who are admitted in an intensive care unit [19].

None of the patients we evaluated showed relevant changes in their blood tests that justified these symptoms and there were no pathological findings in the imaging studies. Moreover, inflammatory markers such as ferritin only showed a slight increase in 25% of patients, in contrast with patients with severe COVID-19.

SARS-COV-2 binds with different cells in the human body. Thus, COVID-19 should be seen as a systemic infection rather than just a respiratory syndrome [20]. Several studies have reported the presence of SARS-CoV-2 RNA in plasma of critical patients, although it has not been detected in non-severe patients [21]. Recently, SARS-CoV-2 RNA has been documented in blood donors [22]. In our cohort, we detected SARS-CoV-2 RNA in plasma in almost half of the patients, and a small proportion of these patients were positive in stool and urine. These patients were discharged or not hospitalized at evaluation and only 24% had suffered severe COVID-19. Median number of days until the first RT-PCR on specimens other than respiratory samples was 55, and in four patients, RT-PCR was positive in plasma for more than 70 days after diagnosis. These findings raise the question of whether these patients may have a persistent systemic viremia and if there is a viral reservoir where SARS-CoV-2 is capable of evading the immunological response of the host, considering that 85% of patients in the cohort showed humoral immune response to SARS-COV-2.

SARS RNA has been detected in extra-respiratory samples (plasma, stool, urine) in a cohort of convalescent patients, weeks after diagnosis (up to 7 weeks in plasma in one patient) and presence of viable virus detected in urine and stools for up to 4 weeks [23]. Moreover, feline coronavirus, an alphacoronavirus distributed worldwide that produces intestinal symptoms and systemic syndromes in cats and some wild felines, may last in feces for months, while in some cats, recurrent/intermittent shedding may occur [24, 25]. Viremia in feline coronavirus seems to decrease during the course of the infection, although some animals show recurrent viremia. Necropsy results in cats with undetectable viremia show the presence of feline coronavirus RNA in a wide range of organs, particularly the colon, liver and mesenteric lymph nodes [26].

As described above, positive results in our cohort were obtained more frequently in plasma samples than in stool or urine. Median Ct values in patients with positive plasma RNA was 36, which is close to the limit of detection of the assay. Moreover, we only obtained this sensitivity with a specific nucleic acid diagnostic kit. Thus, sensitivity limitations of the different RT-PCR kits may explain why viremia was not detected in all patients despite reporting similar symptoms.

Most patients received a combination of antiviral treatment, lopinavir/ritonavir and hydroxychloroquine. No decrease in mortality was seen with the administration of lopinavir/ritonavir in a randomized clinical trial of COVID-19 patients [27]; however, the study included only severe patients and these findings cannot be extrapolated to our context [28, 29]. Recently, two randomized clinical trials have published their results, the RECOVERY trial a randomized open clinical trial who did not find any benefit in 28-days mortality rate in hospitalized COVID-19 patients who received lopinavir/ritonavir [30]; and the MIRACLE trial a double blind randomized clinical trial exploring the combination of lopinavir/ritonavir and interferon Beta-1b in the treatment of Middle East Respiratory Syndrome (MERS) that demonstrated a lower mortality with this combination, especially if started in the first 7 days of symptoms [31]. A randomized SARS-CoV-2 post-exposure prophylaxis trial with hydroxychloroquine, probably the best scenario for a drug, did not reduce the rate of infections in exposed patients [32]. Currently there is an ongoing clinical trial exploring lopinavir/ritonavir monotherapy in COVID-19 patients as an early treatment in the outpatient setting prior to hospitalization (NCT04372628) [33].

The functional scale of patients who received treatment improved in comparison to untreated subjects. However, there is recurrence of symptoms in some patients who received a short-course treatment (14 days), so treatment duration seems a relevant issue.

There are important limitations to this observational study. Viremia was not assessed in patients asymptomatic after been diagnosed with COVID-19. Thus, we do not know if the viremia also persists in asymptomatic patients, in which case symptoms may not be related to viremia. Patients received treatment outside a clinical trial setting with the absence of a placebo-controlled group; therefore, improvements seen in treated patients should not be interpreted as related to the treatment. Treatment bias, in favour of patients with the worst functional status, is another relevant limitation, i.e., patients that will probably have better outcomes because self-reported differences between symptoms will be greater. To assess functional outcomes, we applied a functional scale that has not been validated for patients with the characteristics of this cohort. Finally, we detected RNA but were not able to perform cultures to assess viral viability.

In conclusion, our results suggest a pattern of persistent or recurrent/intermittent SARS-CoV-2 viremia in some patients, causing a clinical curse of non-specific symptoms associated to relevant functional limitations. Further studies in larger series are needed to confirm this hypothesis of persistent viremia in order to avoid diagnosing a great number of patients of chronic fatigue-like syndrome, a disease with poor clinical outcomes. These patients may benefit from antiviral treatment an issue that should be evaluated in randomized placebo-controlled trials.

## Data Availability

The datasets generated during and/or analyzed during the current study area available from the corresponding author on reasonable request.

## Abbreviations

RT-PCR: Reverse transcription polymerase chain reaction
SARS-CoV-2: Severe acute respiratory syndrome coronavirus 2
RNA: Ribonucleic acid
COVID-19: Coronavirus disease 2019
ACE2: Angiotensin-converting enzyme 2 receptor
Ct: Cycle threshold
SARS: Severe acute respiratory syndrome

## References

[1] Johns Hopkins Coronavirus Center. https://coronavirus.jhu.edu/map.html.

[2] Neufel KJ, Leoutsakos JMS, Yan H (2020). Fatigue symptoms during the first year following ARDS. Chest.

[3] Lam MHB, Wing YK, Yu MWM (2009). Mental morbidities and chronic fatigue in severe acute respiratory syndrome survivors. Arch Intern Med.

[4] Tso EYK, Tsang OT, Choi KW (2004). Persistence of physical symptoms in and abnormal laboratory findings for survivors of severe acute respiratory syndrome. Clin Infect Dis.

[5] Moldofsky H, Patcai J (2011). Chronic widespread musculoskeletal pain, fatigue, depression and disordered sleep in chronic post-SARS syndrome; a case- controlled study. BMC Neurol.

[6] Leow MKS, Kwek DSK, Ng AWK (2005). Hypocortisolism in survivors of severe acute respiratory syndrome (SARS). Clin Endocrinol (Oxf).

[7] Zhou P, Yang XL, Wang XG (2020). A pneumonia outbreak associated with a new coronavirus of probable bat origin. Nature.

[8] Wan Y, Jian S, Graham R (2020). Receptor recognition by the novel coronavirus from Wuhan: an analysis based on decade-long structural studies of SARS coronavirus. J Virol.

[9] Wang Q, Zhang Y, Wu L (2020). Structural and functional basis of SARS-CoV-2 entry by using human ACE2. Cell.

[10] Ghlebaw M, Wabg K, Viveiros A (2020). Angiotensin-converting enzyme 2: SARS-CoV-2 receptor and regulator of the renin-angiotensin system. Circ Res.

[11] Heinrich F, Nentwich MF, Bibiza-Freiwald E (2021). SARS-CoV-2 blood RNA predicts outcome in critically ill COVID-19 patients. Open Forum Infect Dis.

[12] Davido B, Seang S, Tubiana R, de Truchis P (2020). Post-COVID-19 chronic symptoms: a post-infectious entity?. Clin Microbiol Infect.

[13] Klok FA, Boon G, Barco S (2020). The post-COVID-19 functional status scale: a tool to measure functional status over time after COVID-19. Eur Respir J.

[14] Carfi A, Bernabei R, Landi F (2020). Persistent symptoms in patients after acute COVID-19. JAMA.

[15] Garrigues E, Janvier P, Kherabi Y, Le Bot A, Hamon A, Gouze H (2020). Post-discharge persistent symptoms and health-related quality of life after hospitalization for COVID-19. J Infect.

[16] Lu X, Schneider E, Jain S (2017). Rhinovirus viremia in patients hospitalized with community-acquired pneumonia. J Infect Dis.

[17] De Jong MD, Simmons CP, Thanh TT (2006). Fatal outcome of human influenza A (H5N1) is associated with high viral load and hypercytokinemia. Nat Med.

[18] Barberis E, Vanella VV, Falasca M (2021). Circulating exosomes are strongly involved in SARS-CoV-2 infection. Front Mol Biosci.

[19] Wintermann GB, Rosendahl J, Weidner K (2018). Self-reported fatigue following intensive care of chronically critically ill patients: a prospective cohort study. J Intensive Care.

[20] Gupta A, Madhavan MV, Sehgal K (2020). Extrapulmonary manifestations of COVID-19. Nat Med.

[21] Zheng S, Fan J, Yu F (2020). Viral loads dynamics and disease severity in patients infected with SARS-CoV-2 in Zhejiang province, China, January–March 2020: retrospective cohort study. BMJ.

[22] Chan L, Zhao L, Gong H (2020). Severe acute respiratory syndrome coronavirus 2 RNA in blood donations. Emerg Infect Dis.

[23] Xu D, Zhang Z, Jin L, Chu F, Mao Y, Wang H (2005). Persistent shedding of viable SARS-CoV in urine and stool of SARS patients during the convalescent phase. Eur J Clin Microbiol Infect Dis.

[24] Tekes G, Thiel HJ (2016). Feline coronavirus: pathogenesis of feline infectious peritonitis. Adv Virus Res.

[25] Pedersen NC, Allen CE, Lyons LA (2008). Pathogenesis of feline enteric coronavirus infection. J Feline Med Surg.

[26] Kipar A, Meli ML, Baptiste KE (2010). Sites of feline coronavirus persistence in healthy cats. J Gen Virol.

[27] Cao B, Wang Y, Wen D (2020). A trial of lopinavir-ritonavir in adults hospitalized with severe COVID-19. N Engl J Med.

[28] Meini S, Pagotto A, Longo B, Vendramin I, Pecori D, Tascini C (2020). Role of lopinavir/ritonavir in the treatment of Covid-19: a review of current evidence, guideline recommendations, and perspectives. J Clin Med.

[29] Klement-Frutos E, Burrel S, Peytavin G (2020). Early administration of ritonavir-boosted lopinavir could prevent severe COVID-19. J Infect.

[30] RECOVERY Collaborative Group (2020). Lopinavir-ritonavir in patients admitted to hospital with COVID-19 (RECOVERY): a randomized, controlled, open-label, platform trial. Lancet.

[31] Arabi YM, Asiri AY, Assiri AM (2020). Interferon beta-1b and lopinavir-ritonavir for middle east respiratory syndrome. N Engl J Med.

[32] Boulware DR, Pullen MF, Bangdiwala AS (2020). A randomized trial of hydroxychloroquine as postexposure prophylaxis for COVID-19. N Engl J Med.

[33] Trial of early therapies during non-hospitalized outpatient window for COVID-19 (TREATNOW). https://clinicaltrials.gov/ct2/show/NCT04372628?term=lopinavir&cond=Coronavirus&draw=3&rank=18. 10.1186/s13063-022-06213-z PMC8990452 35395957

